# Chemosensory learning and memory

**DOI:** 10.3389/fnsys.2012.00073

**Published:** 2012-10-25

**Authors:** Milagros Gallo, Edmund Rolls

**Affiliations:** ^1^University of GranadaGranada, Spain; ^2^Oxford Centre for Computational NeuroscienceOxford, UK

The aim of this issue is to present an updated view of present knowledge and questions raised in the rapidly expanding field of chemosensory (taste and olfactory) learning. Taste is a powerful primary (unlearned) reinforcer, and topics such as olfactory-taste and visual taste association learning are covered in this issue. But the reinforcing properties of taste can themselves be modified, for example, by post-ingestive consequences, for example, in taste aversion learning, and this type of learning is also covered in this issue.

In fact, research on the chemosensory systems has played an important role in advancing knowledge of the brain mechanisms of learning and memory. A well-known example is conditioned taste aversion (CTA). Since the time it was discovered (García et al., 1955), the unique nature of CTA presented a challenge to the contemporary learning theory, and CTA contributed to present theoretical views of learning. CTA learning also became a useful tool for researchers on the neural mechanisms of learning and memory. Jan Bures was a leader in research on the brain mechanisms of CTA in Europe for several decades. We would like to dedicate this special issue to Jan Bures, who passed away on August 24, 2012, in Prague. The field of chemosensory learning is greatly saddened by the news (http://www.ctalearning.com/announcements.asp). He, together with his wife Olga Buresova, was a pioneer during the seventies in applying reversible brain inactivation techniques in order to identify the specific role of the areas forming the CTA circuits. Among other findings, he discovered the critical role of the parabrachial area in taste-visceral signal association, and the relevance of cortico-pontine connections in taste processing (Bures et al., 1998). In addition to his outstanding scientific contributions, Jan was a wonderful colleague and mentor for us and many of the contributors to the present issue and we will never forget him.

The papers forming this issue are representative of the long history and great development of the field thanks to the use of different species and a variety of technical and theoretical approaches. The widely ranging review by Yamamoto and Ueji (2011) of flavor learning including both learned food preferences and aversions, and the paper by Scott (2011) reviewing classic knowledge on the brain mechanisms of CTA, highlight the advances in the field during the last decades. Wider and more complex brain systems than previously thought contribute to flavor learning, with age-dependent interactions between areas such as the insular cortex, amygdala, hippocampal, thalamic, and reward systems (Gámiz and Gallo, 2011). The evidence reported by Neseliler et al. (2011) using an *in vivo* genetically modified rodent model of hypercholinergic innervation is an example of the value of new approaches to support the hypothesis linking acetylcholine and CTA. As Guzmán-Ramos and Bermúdez-Rattoni (2011) describe, major research advances have been made on the cascade of molecular changes involved in the consolidation of CTA taking place during the post-acquisition period.

Remarkable progress has also been made in the field of food preferences. de Araujo (2011) provides a review that includes data obtained both in rodents and Drosophila on the role of taste and energy-sensing systems receiving gastrointestinal and post-absorptive signals in the formation of long-lasting preferences mediated by dopamine release. The elegant experimental work using a variety of techniques reported by Oliveira-Maia et al. (2012) adds evidence on this topic demonstrating the role of the insular cortex in detecting the postingestive effects of sucrose intake.

Closely linked to taste learning in detecting chemical molecules is olfactory learning. As Sandoz (2011) shows in his review, the honeybee has been a model for applying behavioral, neurophysiological and neuroanatomical techniques to research on olfactory learning. Two separate models of the role in olfactory learning of the rat olfactory bulb (Auffarth et al., 2011) and the human glomerulus (Schaefer and Margrie, 2012) are presented.

Finally, Rolls (2011) reviews evidence from primates including humans on the value of taste as a primary reinforcer and the role of the orbitofrontal cortex in building olfactory-taste, and visual-taste associations. He also shows how top–down cognition and attention influence taste and olfactory processing in ways that must involve learning, and also considers the cortical mechanisms involved in taking decisions about olfactory and taste stimuli.
